# Supplementary material for: The Scalable Solid-State Synthesis of a Ni5P4/Ni2P–FeNi Alloy Encapsulated into a Hierarchical Porous Carbon Framework for Efficient Oxygen Evolution Reactions
Source: Nanomaterials (Basel). 2022 May 28;12(11):1848. doi: 10.3390/nano12111848 (PMC9182157; doi:10.3390/nano12111848)
Supplement: Supplementary file 1 [file nanomaterials-12-01848-s001.zip › nanomaterials-1735611-supplementary.pdf]

nanomaterials

Supplementary Material

The Scalable Solid–State Synthesis of a Ni_5P_4/Ni_2P–FeNi Alloy Encapsulated into a Hierarchical Porous Carbon Framework for Efficient Oxygen Evolution Reactions

Xiangyun Tian [1], Peng Yi [1], Junwei Sun [2], Caiyun Li [1], Rongzhan Liu [1,3,*] and Jian–Kun Sun [2,*]

1 College of Textiles and Clothing, Qingdao University, Qingdao 266071, China; txy3287425946@163.com (X.T.); yp13792668731@163.com (P.Y.); 17864235865@163.com (C.L.)
2 College of Chemistry and Chemical Engineering, Qingdao University, Qingdao 266071, China; sunjunwei121@163.com
3 Collaborative Innovation Center for Eco–Textiles of Shandong Province and the Ministry of Education, Qingdao University, Qingdao 266071, China
* Correspondence: qdulrz760504@163.com (R.L.); sunjk@qdu.edu.cn (J.–K.S.)

1. Experimental section

1.1. Peparation of the S–NaCl, S–$NaHCO_3$ and M–NaCl/$NaHCO_3$ catalysts.

Sole NaCl (5.627 mmol), sole $NaHCO_3$ (3.807 mmol) and the mixture of NaCl (5.627 mmol) and $NaHCO_3$ (3.807 mmol) were respectively grinded with $Fe(NO_3)_3{\cdot}6H_2O$ (0.625 mmol), $Ni(NO_3)_2{\cdot}6H_2O$ (1.876 mmol) and chitosan (1 g) in an agate mortar to a homogenous mixture. Then, these mixture were calcined at 700 °C at Argon atmosphere. After rinsing repeatedly with deionized water and ethanol, followed by drying at 60 °C for 2 h, the final pyrolysis products were labeled as S–NaCl, S–$NaHCO_3$, M–NaCl/$NaHCO_3$, respectively.

1.2. Preparation of the Ni_5P_4/Ni_2P@C.

$NiCl_2{\cdot}6H_2O$ (1.876 mmol), Na_2CO_3 (4.717 mmol) and chitosan (1 g) were vigorously grinded in an agate mortar to a homogenous mixture. Then the powders were annealed at 700˚C for 2 h in Argon atmosphere, the carbonized samples have been fully washed and dried (Ni@C). Following the classic phosphating method, Ni@C furnace maintained at 350 ˚C for 2 h under Argon atmosphere at a heating rate of 5 °C min^{-1}. The final black products were denoted as Ni_5P_4/Ni_2P@C.

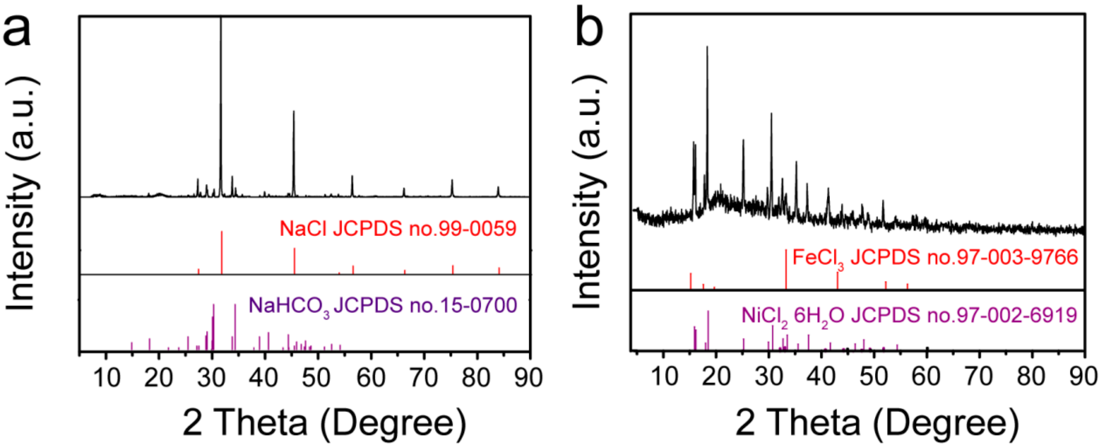

Figure S1. XRD patterns of the solid grinding product based on the precursors with the Na_2CO_3 (**a**), or without the Na_2CO_3 addition (**b**).

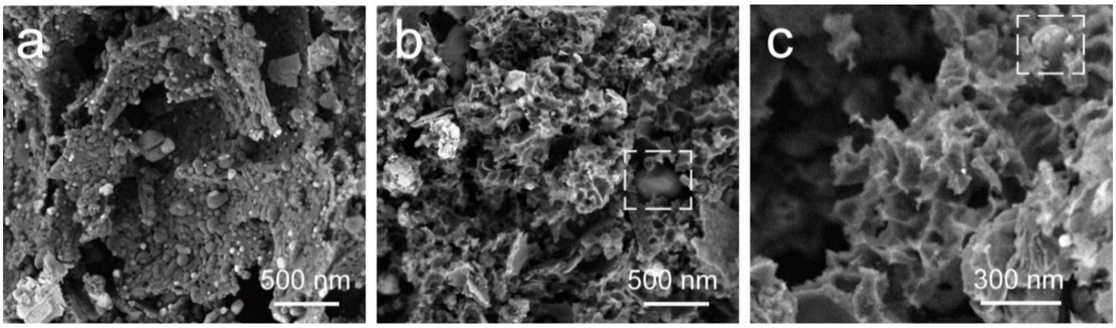

Figure S2. SEM images of pyrolysis product. (**a**) S−NaCl, (**b**) S−$NaHCO_3$ and (**c**) M−NaCl/$NaHCO_3$.

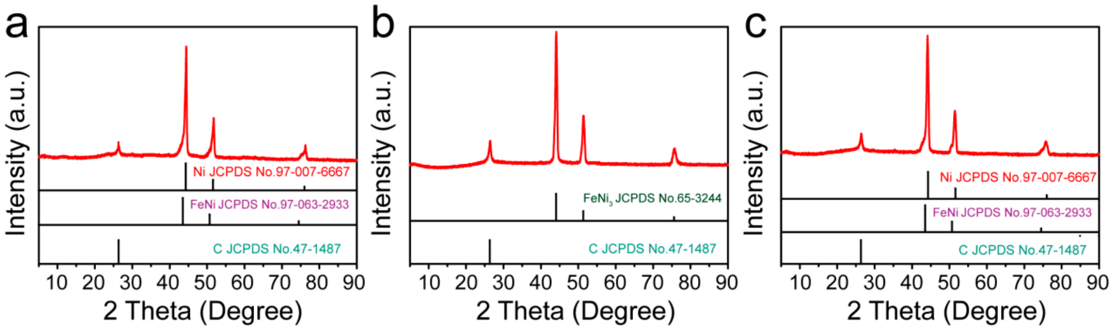

Figure S3. XRD patterns of pyrolysis product. (**a**) S−NaCl, (**b**) S−$NaHCO_3$ and (**c**) M−NaCl/$NaHCO_3$.

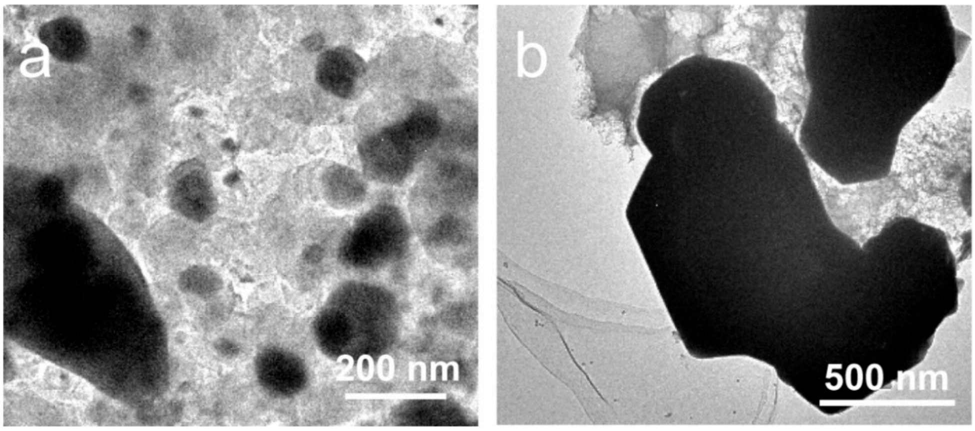

Figure S4. Low−magnification TEM images of (**a**) Ni−FeNi@C and (**b**) $FeNi_3$@AC sample.

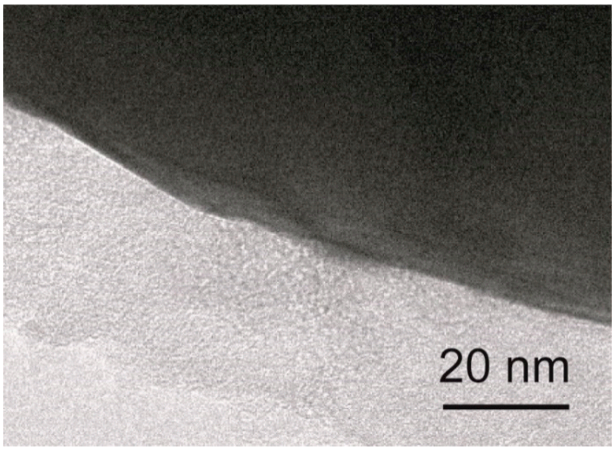

Figure S5. HRTEM image of $FeNi_3$@AC sample.

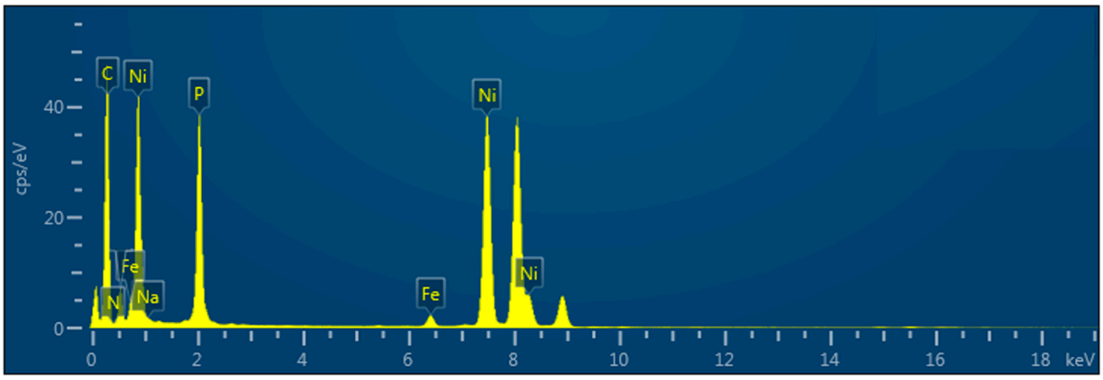

Figure S6. EDS spectrum of Ni_5P_4/Ni_2P−FeNi@C.

Table S1. Elemental contents of the Ni_5P_4/Ni_2P−FeNi@C sample.

Elements	wt %	atomic percentage
C	42.60	70.73
P	18.94	12.19
Fe	6.02	6.06
Ni	32.44	11.02

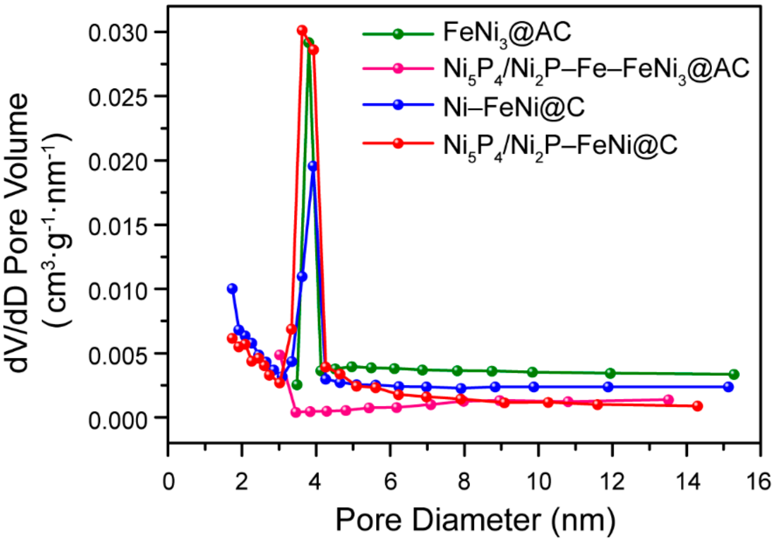

Figure S7. Pore size distribution curves of $FeNi_3$@AC, Ni−FeNi@C, Ni_5P_4/Ni_2P−Fe−$FeNi_3$@AC and Ni_5P_4/Ni_2P−FeNi@C samples.

Table S2. Comparison of surface area properties of as−prepared catalysts.

Samples	BET Surface Area (m^2/g)	BJH Desorption average pore diameter (nm)	BJH Desorption cumulative volume of pores between 17.000 Å and 3,000.000 Å diameter (cm^3/g)
$FeNi_3$@AC	143.32	16.24	0.19
Ni_5P_4/Ni_2P−Fe−$FeNi_3$@AC	12.12	20.48	0.09
Ni−FeNi@C	182.12	14.62	0.21
Ni_5P_4/Ni_2P−Fe Ni@C	82.13	8.23	0.10

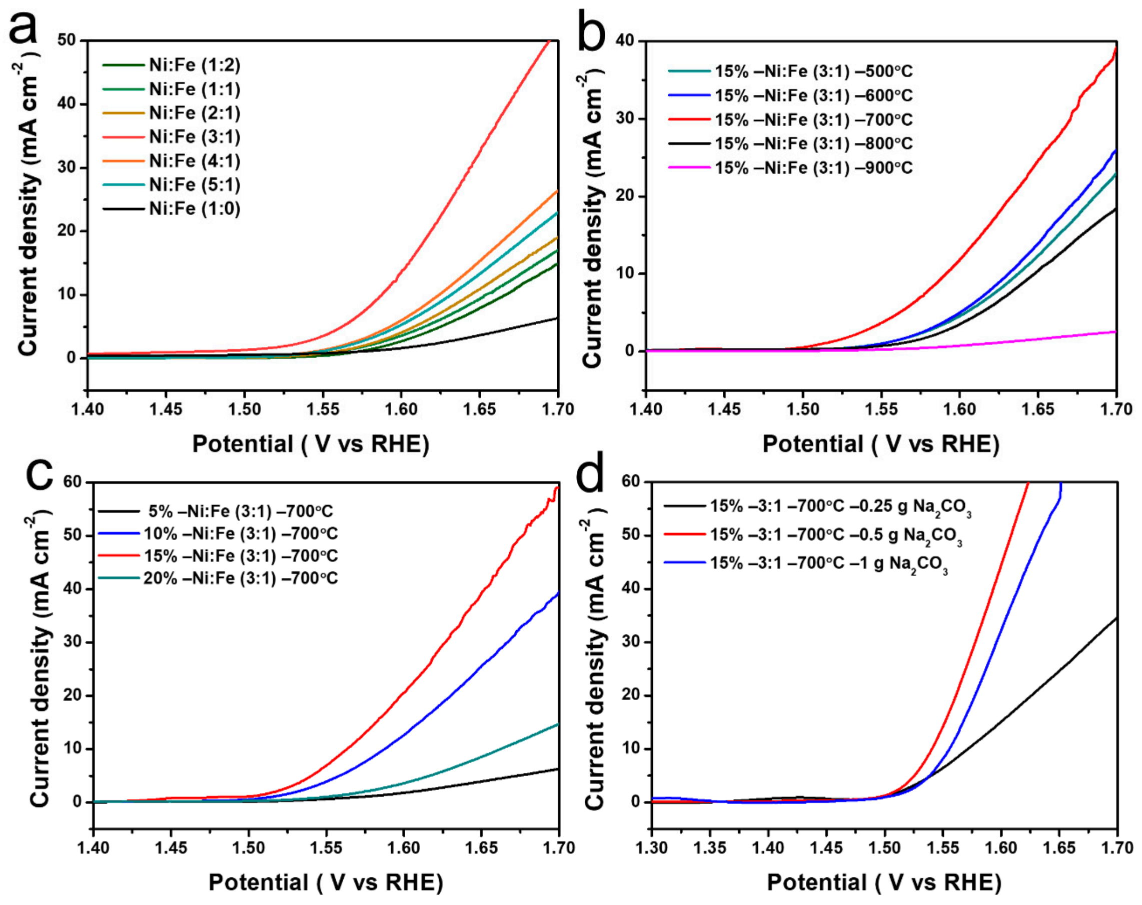

Figure S8. The OER LSV curve of Ni_5P_4/Ni_2P−FeNi@C catalyst based on different synthesis conditions. (**a**) The ratio of nickel to iron. (**b**) The pyrolysis temperature. (**c**) The total amount of metals. (**d**) The amount of Na_2CO_3 addition.

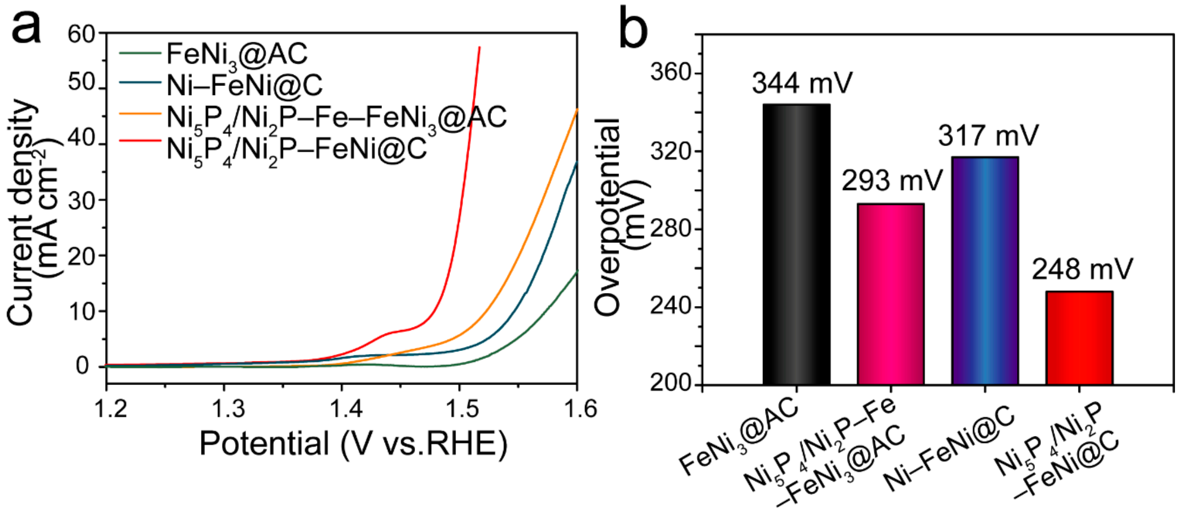

Figure S9. (**a**) 85% iR−corrected OER LSV curves of $FeNi_3$@AC; Ni−FeNi@C; Ni_5P_4/Ni_2P−Fe−$FeNi_3$@AC and Ni_5P_4/Ni_2P−FeNi@C samples prepared by balling−milling methods in 1 M KOH electrolyte. (**b**) The comparative overpotentials of different catalysts at a current density of 10 mA cm^{-2}.

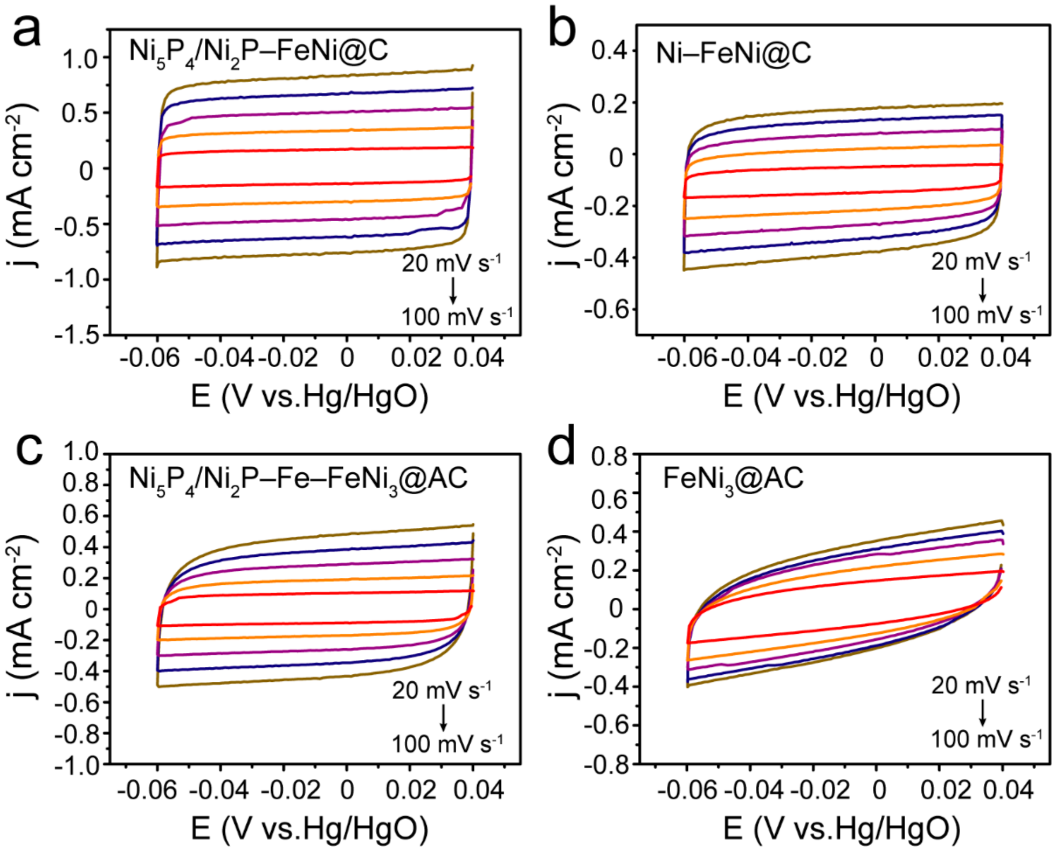

Figure S10. CV curves versus scan rates for different electrode catalysts at scan rates ranging from 20 mV s^{-1} to 100 mV s^{-1} with an interval point of 20 mV s^{-1}. (**a**) Ni_5P_4/Ni_2P−FeNi@C; (**b**) Ni−FeNi@C; (**c**) Ni_5P_4/Ni_2P−Fe−$FeNi_3$@AC and (**d**) $FeNi_3$@AC.

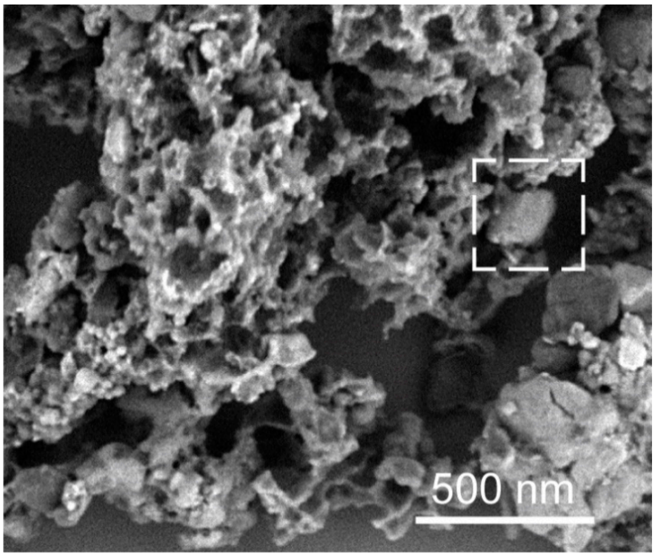

Figure S11. SEM image of Ni_5P_4/Ni_2P−FeNi@C after 140 h stability test at 20 mA cm^{-2}.

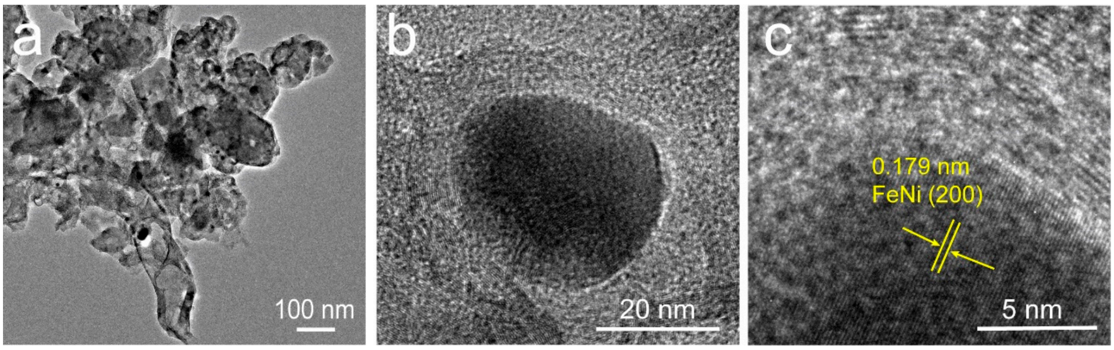

Figure S12. (**a**) TEM and (**b–c**) HRTEM images of Ni_5P_4/Ni_2P−FeNi@C sample after 140 h stability test at 20 mA cm$^{-2.}$

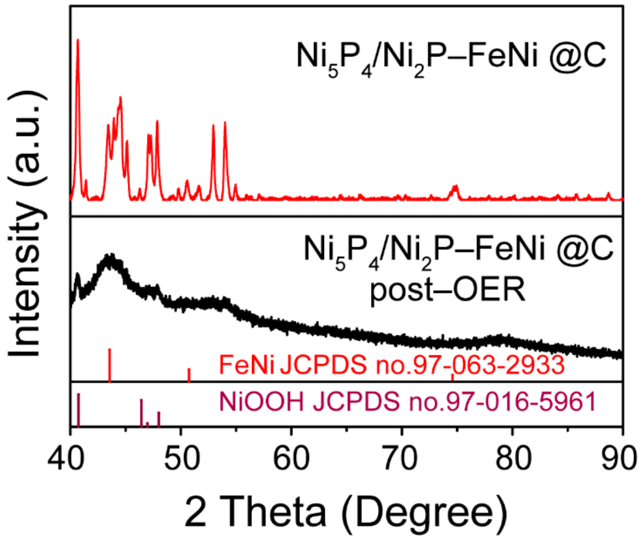

Figure S13. XRD patterns of the initial and post−OER Ni_5P_4/Ni_2P−FeNi@C sample.

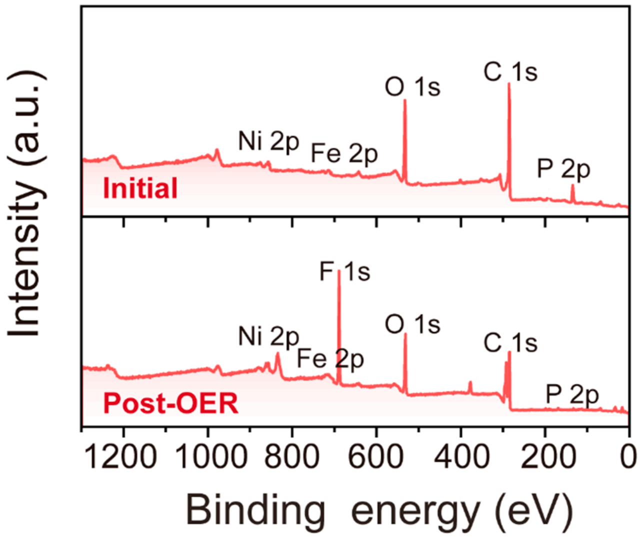

Figure S14. XPS survey spectrum of Ni_5P_4/Ni_2P−FeNi@C sample before and after OER process.
